# Proximal Tubular Injury in Medullary Rays Is an Early Sign of Acute Tacrolimus Nephrotoxicity

**DOI:** 10.1155/2015/142521

**Published:** 2015-06-24

**Authors:** Diane Cosner, Xu Zeng, Ping L. Zhang

**Affiliations:** ^1^Department of Anatomic Pathology, William Beaumont Hospital, Royal Oak, MI 48073, USA; ^2^Department of Pathology, Temple University Hospital, Philadelphia, PA 19140, USA

## Abstract

Tacrolimus (FK506) is one of the principal immunosuppressive agents used after solid organ transplantations to prevent allograft rejection. Chronic renal injury induced by tacrolimus is characterized by linear fibrosis in the medullary rays; however, the early morphologic findings of acute tacrolimus nephrotoxicity are not well characterized. Kidney injury molecule-1 (KIM-1) is a specific injury biomarker that has been proven to be useful in the diagnosis of mild to severe acute tubular injury on renal biopsies. This study was motivated by a patient with acute kidney injury associated with elevated serum tacrolimus levels in whom KIM-1 staining was present only in proximal tubules located in the medullary rays in the setting of otherwise normal light, immunofluorescent, and electron microscopy. We subsequently evaluated KIM-1 expression in 45 protocol and 39 indicated renal transplant biopsies to determine whether higher serum levels of tacrolimus were associated with acute segment specific injury to the proximal tubule, as reflected by KIM-1 staining in the proximal tubules of the cortical medullary rays. The data suggest that tacrolimus toxicity preferentially affects proximal tubules in medullary rays and that this targeted injury is a precursor lesion for the linear fibrosis seen in chronic tacrolimus toxicity.

## 1. Introduction

Deterioration of renal function with tacrolimus (FK506) therapy has been widely reported in adult patients with normal renal function who underwent liver transplantation [1, 2]. A striped pattern of medullary ray fibrosis (also called linear fibrosis) has been described in the kidneys of patients undergoing long-term treatment with the calcineurin inhibitors, cyclosporine, or tacrolimus [3–5]. Calcineurin inhibitors can cause this pattern of injury in animals, possibly contributing to the relatively low availability of oxygen in the medullary rays [6, 7]. Early diagnosis of acute tacrolimus nephrotoxicity in humans prior to fibrosis may better guide evaluation of impaired renal function and serve to justify modification of dosing of the agent. There is currently no consistently recognized pathological pattern of acute tacrolimus nephrotoxicity.

KIM-1 is a type I transmembranous protein whose expression is undetectable in normal renal tissue but is markedly upregulated with injury of proximal tubule epithelial cells in rats [8] and is upregulated in human biopsies with tubular injury [9, 10]. KIM-1 is a receptor involved in phagocytosis and internalization of apoptotic bodies and necrotic debris in injured kidneys; thus it involves tubular repairing during acute tubular injury [11]. Motivated by a case of acute tacrolimus renal injury in a patient status after liver transplant that was characterized by dominant expression of KIM-1 in the proximal tubules of the medullary rays, we evaluated whether early proximal tubule injury could be identified by KIM-1 expression in the medullary rays in acute nephrotoxicity from tacrolimus.

## 2. Methods

In the initial case, frozen sections of the renal biopsy were cut for direct immunofluorescent stains of IgG, IgA, IgM, C3, C1q, kappa, lambda, and albumin, using a Dako Autostainer (Carpinteria, CA). The remaining renal tissue was divided into two parts and fixed with 10% formalin for light microscopy and 3% glutaraldehyde for electron microscopy, respectively. Formalin fixed tissue was embedded, sectioned, and examined using Hematoxylin-Eosin (HE), Periodic Acid Schiff (PAS), and Masson's trichrome for light microscopy. After fixation, tissue for electron microscopy was routinely postfixed in osmium tetroxide, embedded in resin, sectioned, and stained with uranyl acetate and lead citrate. Tissues placed on grids were examined using a transmission electron microscope. Slides for light microscopy were treated for 20 minutes using a heat-induced antigen retrieval protocol (Target Retrieval Solution, DakoCytomation, Carpinteria, CA). A mouse monoclonal antibody (AKG7), directed against the ectodomain of human KIM-1 [10], was used as the primary antibody in this study. AKG7 antibody (diluted 1 : 8, kindly provided by Dr. Joseph V. Bonventre, Renal Division, Brigham and Women's Hospital, Boston) was applied for one hour, peroxidase-labeled goat anti-mouse secondary antibody (Env+ kit, DakoCytomation) for 30 minutes, and the chromagen DAB (3,3′-diaminobenzidine, Env+ kit, DakoCytomation) for ten minutes to achieve a brown KIM-1 stain in the proximal tubules.

To extend our observation that KIM-1 may be helpful in identifying acute tacrolimus nephrotoxicity, we evaluated 45 protocol renal transplant biopsies and 39 indicated renal transplant biopsies with acute tubular injury from patients maintained on tacrolimus treatment from 2005 to the end of 2007 (the study protocol was proven by the Institute Research Board). In both protocol biopsies and indicated biopsies, any cases with significant inflammation (related to subclinical acute cellular rejection or acute cellular rejection) were excluded from the study because we wanted to focus on noninflammatory injury. KIM-1 protein expression was evaluated in each biopsy as previously described [10]. KIM-1 staining was scored manually for all of the biopsy cases. The staining intensity scores of epithelial cells were graded from 0 to 3+ (0: no staining; +/− [0.5]: focal weak fine granular staining; 1+: more widespread weak fine granular staining; 2+: moderate granular staining; and 3+: strong large granular staining). Serum creatinine and tacrolimus levels were determined from the medical record. Results were expressed as the mean ± SEM. Data among the three groups were compared using an ANOVA test. A *p* value less than 0.05 was considered significantly different.

## 3. Results

### 3.1. Initial Case

**Figure 1:**
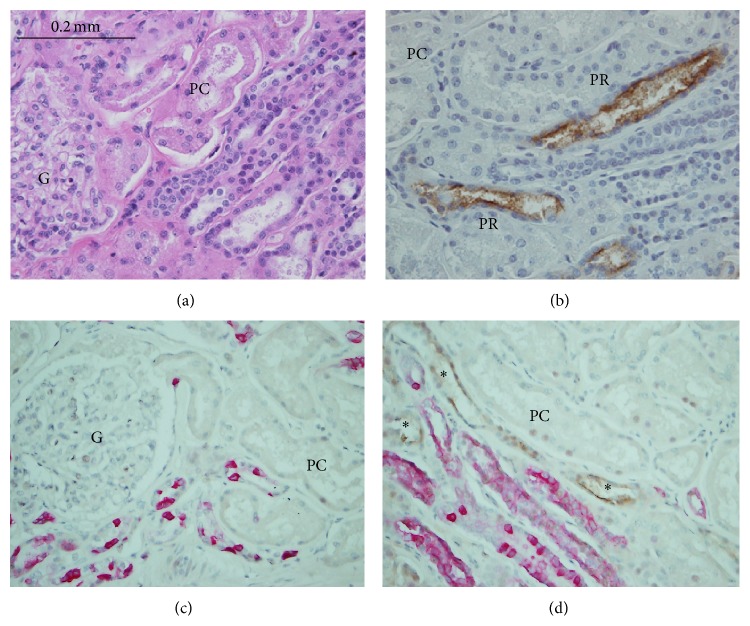
Acute tacrolimus nephrotoxicity, identified by KIM-1 staining in the case. On routine light microscopy, the renal tissue appears normal in pars convoluta (PC) and medullary rays located in the right lower corner of A (H&E staining). However, KIM-1 staining (B) revealed 2+ positive staining (brown color) reflective of acute kidney injury in the proximal tubules located in medullary rays (pars recta, PR) but not in the proximal tubules around glomeruli (PC), indicating acute tubular injury, a pattern consistent with acute nephrotoxicity of tacrolimus. KIM-1 and cytokeratin-7 (a distal tubular marker) coexpression is shown in C and D. In both panel C and panel D, pink stained tubules were distal nephron tubules. In panel C, PC around glomerulus stained negatively for KIM-1 and in panel D, PR in medullary rays stained positively for KIM-1 (brown color staining, indicated by asterisk). Magnification ×400 for A-B and ×200 for C-D.

A 45-year-old man, with liver failure due to hepatoma and alcoholic cirrhosis and with normal renal function, underwent orthotopic liver transplantation. He received a postmortal liver transplant and was on maintenance treatment with tacrolimus at 4 mg twice a day, mycophenolate mofetil 500 mg twice a day, and prednisone 20 mg daily. Following liver transplantation his liver function returned to normal (Table 1). A month following the liver transplantation, he presented with acute kidney injury with a serum creatinine of 3 mg/dL. His serum tacrolimus level was found to be acutely elevated at 28 ng/dL from a maintenance level of about 8 ng/dL. He underwent a kidney biopsy to identify the cause of his acute kidney injury. By light microscopy, nineteen normal glomeruli were present without thrombi, diffuse proliferation, or crescent formation noted in the two renal cortical tissues submitted. No medulla was present in the submitted renal tissue. There was no interstitial nephritis, acute pyelonephritis, or vasculitis. Proximal tubules around the glomeruli maintained brush borders on PAS stained sections. Proximal tubules in medullary rays were mixed with distal nephron tubules and appeared not remarkable by routine light microscopy. When the tissue was stained for KIM-1, however, the luminal surface of proximal tubules located in medullary rays (pars recta), but not around the glomeruli (pars convoluta), demonstrated positive expression of KIM-1 (Figure 1), suggestive of acute tubular injury from tacrolimus nephrotoxicity. Staining of the glomeruli for IgG, IgA, IgM, C1q, C3, kappa, and lambda was negative by immunofluorescence. Electron microscopy revealed normal thickness of glomerular capillary loops with well-maintained foot processes of glomerular visceral epithelium. No immune complex deposits were ultrastructurally identified in the four glomeruli.

Based on these findings, the tacrolimus dose was reduced and his serum levels returned to therapeutic range. The patient's serum creatinine fell to 1.4 mg/dL over the ensuing 11 days. A year after the liver transplantation, the patient's liver function was normal and his serum creatinine was 1.0 mg/dL (Table 1).

### 3.2. Study of 84 Renal Transplant Biopsies in 82 Patients Taking Tacrolimus

This case motivated us to examine the KIM-1 staining pattern in a total of 84 renal transplant biopsies in 82 patients who had been taking tacrolimus (from 2005 to the end of 2007, blindly evaluated by PLZ only). In 45 cases the biopsies were “protocol biopsies” and the remaining 39 were “indicated” biopsies due to elevation of serum creatinine. The patients were divided into 3 groups, based on KIM-1 staining status. Group 1 (*n* = 13) consisted of patients who showed no KIM-1 staining in the biopsies (13 protocol biopsies, 0 indicated biopsies). Group 2 (*n* = 28) included 22 indicated biopsies and 6 protocol biopsies in which KIM-1 staining was present in most of cortical proximal tubules, both around glomeruli (pars convoluta, also called S1 segment) and in medullary rays (pars recta, also called S2 segment). Group 3 (*n* = 43) consisted of 17 indicated renal biopsies and 26 protocol biopsies that revealed KIM-1 staining only in the proximal tubules located in the medullary rays (pars recta). Two patients in group 3 required repeat biopsies. In the analysis of 45 protocol biopsies and 39 indicated renal transplant biopsies from patients on tacrolimus, the patients with KIM-1 staining in both S1 and S2 segments (group 2) had the highest KIM-1 expression scores and serum creatinine levels (Table 2). The serum levels of tacrolimus, however, were higher in group 3 where KIM-1 staining was localized exclusively to the proximal tubules located in the medullary rays (pars recta) than in either group 1 (no KIM-1 staining) or group 2 (KIM-1 staining in both pars convoluta and pars recta). In 43 (51%) of 84 cases (including both indicated and protocol biopsies) and 17 (44%) out of 39 indicated biopsies, KIM-1 protein expression was seen in proximal tubules of medullary rays (pars recta), suggesting that acute tacrolimus toxicity may be more common than what is currently recognized as a cause of renal injury. In addition, 32 (71%) of 45 protocol biopsies exhibited KIM-1 positive staining (13% in group 2 and 58% in group 3), reflective of ongoing injury (presumably from medications or other etiologies such as immune challenge from recipients), even when serum creatinine levels remain in the normal range.

## 4. Discussion

The precise mechanism of tacrolimus-induced nephrotoxicity is not completely understood. Acute nephrotoxicity has been attributed to hemodynamic changes characterized by renal vasoconstriction that is dose-related and reversible [6]. On the other hand, chronic tacrolimus nephrotoxicity can be progressive and irreversible. Chronic pathologic changes include tubular atrophy, afferent arteriolar hyalinosis, and striped interstitial fibrosis (also called linear fibrosis) with mononuclear cell infiltration [1, 5]. Therefore, early detection of acute tacrolimus nephrotoxicity may preserve kidney function through adequate modification in tacrolimus doses.

**Figure 2:**
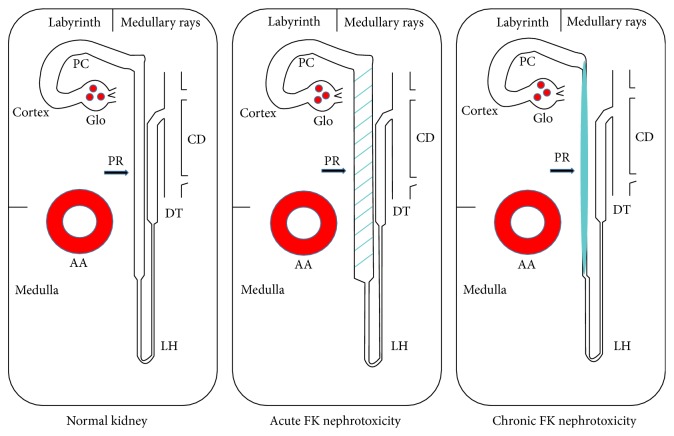
Diagrammatic changes in pars recta (PR) in normal kidney (left panel), in kidney with acute tacrolimus (FK) nephrotoxicity (middle panel) and in kidney with chronic tacrolimus nephrotoxicity (right panel). In this simplified kidney diagram of normal kidney (left panel), the pars convoluta (PC) located in labyrinth of cortex is connected with pars recta (PR) in medullary rays and outer stripe of outer medulla. The latter is further connected with distal nephron tubules including loop of Henle (LH), distal tubules (DT), and collecting duct (CD). The arcuate artery (AA) is located at the cortical-medullary junction. During acute tacrolimus nephrotoxicity (middle panel), PR becomes dilated (dashed segment) that injury can be detected by positive KIM-1 staining. During chronic tacrolimus nephrotoxicity (right panel), the PR becomes atrophic with surrounding striped fibrosis (illustrated with solid bar) along the medullary rays.

It is often difficult to identify tacrolimus toxicity on a renal transplant biopsy especially if the patient is on a number of nephrotoxic drugs. In humans, renal allografts may be affected by many immune and nonimmune factors. Some factors that can change the tacrolimus levels may include compliance of patients to take the medication, dehydration state (summer or winter), renal function to excrete the medications, and body weight of recipients. In addition, many factors may also accelerate the tacrolimus toxicity as each transplant recipient has only one graft (half of normal glomerular filtration rate), and transplant patients have different types of grafts (postmortal donor, donor pathology, living related/nonrelated donor) and their own existing health issues. Serum tacrolimus levels and potentially tacrolimus toxicity are affected by other medications especially some of the antibiotics. As this study indicates, episodes of increased serum levels of tacrolimus can potentially cause acute injury to the renal allograft which may affect the long-term survival of the transplanted kidney. The specific injury caused by elevated tacrolimus levels can be identified by KIM-1 staining of the proximal tubules in the medullary rays (pars recta) of the renal allograft biopsy. Our data with KIM-1 expression in medullary rays (pars recta) in protocol biopsies suggests that tacrolimus nephrotoxicity can be a subclinically insidious process in many patients, which may lead to chronic injury and linear fibrosis in the medullary rays when the drug is taken at maintenance doses. The proximal tubular injury in medullary rays (pars recta), highlighted by KIM-1 expression, may be the precursor lesion of subsequent linear fibrosis characteristically found on renal biopsies of patients with chronic tacrolimus toxicity (Figure 2).

Deterioration of renal function with tacrolimus (FK506) therapy has been widely reported in adult patients with normal renal function who underwent liver transplantation [1, 2]. A striped pattern of medullary ray fibrosis has been described in the kidneys of patients undergoing long-term treatment with the calcineurin inhibitors, cyclosporine, or tacrolimus [3–5]. Calcineurin inhibitors can cause this pattern of injury in animals [6, 7], possibly contributing to the relatively low availability of oxygen in the medullary rays. There is currently no consistently recognized pathological pattern for early diagnosis of acute tacrolimus nephrotoxicity, although thrombotic microangiopathy and osmotic changes in renal tubules can be identified during prominent acute tacrolimus nephrotoxicity. Early diagnosis of acute tacrolimus nephrotoxicity in humans prior to fibrosis may better guide evaluation of impaired renal function and serve to justify modification of dosing of the agent.

KIM-1 is a type I transmembranous protein whose expression is undetectable in normal renal tissue but is markedly upregulated with injury of proximal tubule epithelial cells in rats and humans [8–10]. KIM-1 is a receptor involved in phagocytosis and internalization of apoptotic bodies and necrotic debris in injured kidneys [11]. In our current case, the pars recta in medullary rays was hard to distinguish from the distal nephron tubules by light microscopy, which may result in assuming normal pars recta epithelia. KIM-1 staining, however, was markedly positive in pars recta in medullary rays whereas the pars convoluta did not express this specific injury marker. This index case plus the finding that this specific pars recta staining is correlated with high systemic levels of tacrolimus suggests that KIM-1 expression can highlight subtle tubular injury in medullary rays where vulnerable proximal tubules are located and identify the very early stages of tacrolimus toxicity.

In summary, our initial case is a good self-control study to document acute nephrotoxicity of tacrolimus. This patient has normal renal function before liver transplantation and tacrolimus treatment but he was found to have acute renal failure when his serum tacrolimus level was accumulated following the liver transplantation. When we found acute tubular injury highlighted by KIM-1 expression in the S2 segment of proximal tubules (vulnerable to calcineurin inhibitor toxicity), the treatment with tacrolimus was withdrawn, tacrolimus level was dropped to normal, and patient's renal function returned to normal level again. With additional studies of KIM-1 expression and its close association with the serum levels of tacrolimus in more transplant recipients, we conclude that KIM-1 is a sensitive injury biomarker detecting the early acute tacrolimus nephrotoxicity in proximal tubules. KIM-1 staining can be used to establish the cause of acute kidney injury from this drug by its specific and characteristic staining of the proximal tubules located in the medullary rays (pars recta) in the setting of sparing of the proximal tubules around the glomeruli (pars convoluta). KIM-1 staining may also be an early marker for predicting the subsequent development of the chronic irreversible fibrosis that is known to be associated with tacrolimus therapy, as prolonged KIM-1 presence in the injured kidneys may be associated with increased interstitial fibrosis in a recent study [12]. Recognition of this toxicity can motivate enhanced surveillance and potentially the modification of dosing well before changes in renal function and chronicity become apparent.

## Figures and Tables

**Table 1:** Flowchart of serum tacrolimus levels and clinical hepatic and renal indices in index case.

	12 days before	1 day before	Liver transplant	Days after transplant						
				9	12	34	35 (biopsy day)	41	45	1 year
FK	—	—	3.1	6.4	5.1	9.9	28.0	8.7	6.5	8.8
Cr	0.8	0.7	0.8	0.9	1.6	3.0	2.8	2.4	1.4	1.0
BUN	10	11	32	18	38	59	59	53	22	22
AP	173	188	74	98	—	—	81	223	100	104
AST	79	89	144	16	—	—	21	29	25	32
ALT	58	61	246	61	—	—	9	15	19	46
Total bili.	4.0	3.2	1.4	0.9	—	—	0.3	0.4	0.4	0.5

FK: FK506 (ng/dL); Cr: creatinine (mg/dL); BUN: blood urea nitrogen (mg/dL); AP: alkaline phosphatase (25–125 U/L); AST: aspartate aminotransferase (8–36 U/L); ALT: alanine aminotransferase (8–67 U/L); total bili.: total bilirubin (0.3–1.3 mg/dL). —: not available.

**Table 2:** KIM-1 score, renal functional indices, and tacrolimus levels in three groups of renal transplant biopsies.

	Three groups	KIM-1 scores	Serum Cr (mg/dL)	Serum tacrolimus (ng/dL)
Group 1	Controls (KIM-1 negative) *N* = 13	0.00 ± 0.00	1.24 ± 0.09	8.53 ± 0.76
Group 2	KIM-1 + in all cortical PT *N* = 28	1.86 ± 0.16^*∗*^	4.20 ± 0.49^*∗*^	5.92 ± 0.61^*∗*^
Group 3	KIM-1 + in PT of medullary rays *N* = 43	1.16 ± 0.10^*∗*#^	1.89 ± 0.26^#^	10.85 ± 0.60^*∗*#^

^*∗*^ *p* < 0.05 versus group 1, ^#^ *p* < 0.05 versus group 2; PT: proximal tubules.
